# A molecular chaperone activity of CCS restores the maturation of SOD1 fALS mutants

**DOI:** 10.1038/s41598-017-17815-y

**Published:** 2017-12-12

**Authors:** Enrico Luchinat, Letizia Barbieri, Lucia Banci

**Affiliations:** 1 Magnetic Resonance Centre (CERM), University of Florence, 50019 Sesto Fiorentino, Italy; 2 Department of Biomedical, Clinical and Experimental Sciences, University of Florence, 50134 Florence, Italy; 3 Interuniversity Consortium for Magnetic Resonance of Metallo Proteins (CIRMMP), 50019 Sesto Fiorentino, Italy; 4 Department of Chemistry, University of Florence, 50019 Sesto Fiorentino, Florence Italy

## Abstract

Superoxide dismutase 1 (SOD1) is an important metalloprotein for cellular oxidative stress defence, that is mutated in familiar variants of Amyotrophic Lateral Sclerosis (fALS). Some mutations destabilize the apo protein, leading to the formation of misfolded, toxic species. The Copper Chaperone for SOD1 (CCS) transiently interacts with SOD1 and promotes its correct maturation by transferring copper and catalyzing disulfide bond formation. By *in vitro* and in-cell NMR, we investigated the role of the SOD-like domain of CCS (CCS-D2). We showed that CCS-D2 forms a stable complex with zinc-bound SOD1 in human cells, that has a twofold stabilizing effect: it both prevents the accumulation of unstructured mutant SOD1 and promotes zinc binding. We further showed that CCS-D2 interacts with apo-SOD1 *in vitro*, suggesting that in cells CCS stabilizes mutant apo-SOD1 prior to zinc binding. Such molecular chaperone function of CCS-D2 is novel and its implications in SOD-linked fALS deserve further investigation.

## Introduction

Amyotrophic Lateral Sclerosis (ALS) is a fatal neurodegenerative disease characterized by the death of motor neurons in the brain and spinal cord. Human copper, zinc superoxide dismutase (SOD1) is a conserved intracellular metalloprotein that protects the cell from oxidative damage. It has been shown that SOD1 is implicated in a sizable fraction of the familial variants of ALS (fALS)^1^. Several missense mutations in the *sod1* gene scattered throughout the polypeptide of SOD1 have been correlated with the onset of fALS^2^. These mutations have been linked to the formation of amorphous aggregates enriched in SOD1 in the spinal cord of fALS patients and in transgenic mice^3–6^.

In order to reach its active conformation, SOD1 has to undergo several post-translational maturation events in the cytoplasm, which include zinc binding, dimerization, copper binding and the formation of an intramolecular disulfide bond. All these post-translational events stabilize the fold of SOD1, which in the apo state is intrinsically unstable and prone to unfolding and aggregation^7,8^. Some fALS mutations, termed wild type-like (WT-L), do not affect the enzymatic activity of the mature protein, as they do not perturb the geometry of the SOD1 metal binding sites, nor the disulfide bond^9,10^. These mutations however are known to further destabilize the structure of the apo protein, increasing the fraction of unfolded protein and eventually leading to misfolding and to the formation of the cytotoxic species^7,8,11,12^.

The molecular events leading the mature, enzymatically active form of SOD1 have been extensively studied *in vitro*. Disulfide-reduced, monomeric apo-SOD1 binds zinc spontaneously, and forms a stable homodimer^13,14^. Copper binding and disulfide bond formation are mediated by a specific partner, the Copper Chaperone for SOD1 (CCS)^15,16^. CCS acts as a metallochaperone through its N-terminal Atx1-like domain (D1) and as oxidoreductase through its C-terminal domain (D3), whereas the second, SOD-like domain (D2) is responsible for the interaction with SOD1^15–24^. Recently, the maturation steps of SOD1 have been observed in living human cells^25^ through in-cell NMR^26,27^. It has been shown that some WT-L fALS SOD1 mutants fail to bind zinc when they are overexpressed in the human cytoplasm, and accumulate as unstructured species that may act as precursors in the pathogenic aggregation pathway^28^. Importantly, this mechanism can be reverted by co-expressing CCS, indicating that the copper chaperone is able to pull the nascent SOD1 molecules towards the correct folding and maturation pathway.

Here, we investigated further how CCS exerts this protective role. We characterized by in-cell NMR the interaction between CCS and SOD1, both WT and the WT-L fALS mutants A4V, T54R, G93A, I113T, in the cytoplasm of living human cells. We showed that the SOD-like domain of CCS, D2, acts as a molecular chaperone in the human cytoplasm. D2 alone is sufficient to interact with the immature, metastable state of fALS SOD1 mutants, and effectively prevents the accumulation of unstructured species. In the absence of D1 and D3, the CCS-dependent maturation cannot occur and the SOD1-D2 heterodimer becomes a stable intermediate, whereas full-length CCS interacts transiently with SOD1 allowing its complete maturation. *In vitro* analysis revealed that, in the heterodimer observed in cells, SOD1 is in the zinc-bound form. These data suggest that D2 of CCS favours zinc binding to mutant SOD1 by interacting either with the zinc-bound protein and preventing zinc dissociation, or with the apo protein stabilizing it and allowing zinc binding. In favour of the latter hypothesis, we further showed that D2 of CCS is able to interact with apo-SOD1 *in vitro*, and therefore that CCS acts as a molecular chaperone towards the most immature state of SOD1.

## Results

### CCS-D2 forms a stable heterodimer with zinc-bound WT SOD1

**Figure 1:**
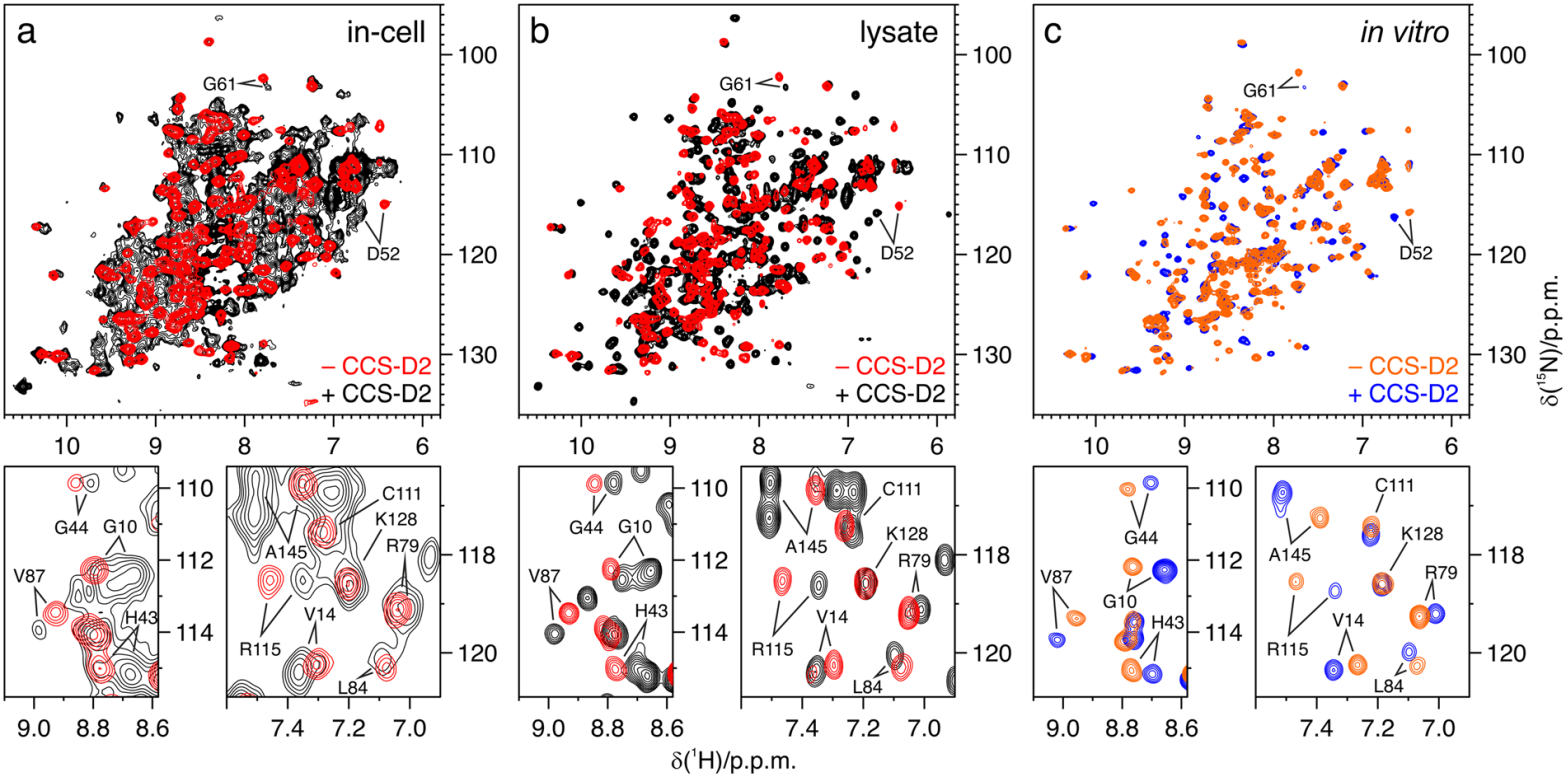
CCS-D2 forms a stable complex with E,Zn-SOD1^SH^ both in cells and *in vitro*. (**a**) ^1^H-^15^N NMR spectra of human cells expressing [U-^15^N]-labelled WT SOD1 either alone (red) or together with [U-^15^N]-labelled CCS-D2 (black). (**b**) ^1^H-^15^N NMR spectra of the corresponding cell lysates. (**c**) *In vitro* ^1^H-^15^N NMR spectra of [U-^15^N]-labelled WT SOD1 without (orange) and with 1 equivalent (blue) of unlabelled CCS-D2. SOD1 signals experiencing chemical shift changes upon complex formation are labelled in the full spectra (upper panels) and in the detailed views (lower panels). See also Supplementary Figures S1 and S2.

WT SOD1 overexpressed in the human cell cytoplasm in the presence of excess zinc is found in a stable intermediate maturation state, i.e. a homodimer with one zinc ion bound to each monomer, and all cysteines reduced (henceforth E,Zn-SOD1^SH^)^25^. This species is free to tumble in the cytoplasm, and gives rise to well resolved signals in the ^1^H-^15^N in-cell NMR spectra (Fig. 1a). When full-length CCS (FL-CCS) is co-expressed at similar levels, SOD1 maturation proceeds further along its pathway: the oxidized zinc-bound SOD1 (E,Zn-SOD1^SS^) is observed in defect of copper, whereas in excess of copper the fully mature protein (Cu,Zn-SOD1^SS^) is observed^25^. CCS is not visible in the ^1^H-^15^N in-cell NMR spectra, with the exception of a few signals (Supplementary Figure S1a), due to interactions with large cellular components such as the plasma membrane^29^, but is clearly observed in the NMR spectra of the cell lysates (Supplementary Figure S1b).

When CCS-D2 was co-expressed with WT SOD1 (95.7 ± 2.6 µM and 118.2 ± 5.4 µM, respectively), the resulting in-cell NMR spectra were remarkably different from those of E,Zn-SOD1^SH^, suggesting the formation of a stable complex between SOD1 and CCS-D2 (Fig. 1a). An overall line broadening was observed, and a few signals of SOD1 were shifted. Several more signals arising from CCS-D2 were also present. The line broadening observed in the in-cell NMR spectra likely arises from the residual interactions of the CCS-D2 monomer with the plasma membrane; consistently, CCS-D2 expressed alone was not detected by in-cell NMR (Supplementary Figure S1c,d) due to such interactions. Upon cell lysis, the complex between SOD1 and CCS-D2 remained unaltered and gave rise to sharp signals in the lysate NMR spectra, with no obvious chemical shift changes with respect to the in-cell NMR spectra (Fig. 1b).

Comparison with *in vitro* NMR spectra acquired on [U-^15^N]-E,Zn-SOD1^SH^ titrated with 1 equivalent of unabelled CCS-D2 (Fig. 1c) confirmed that the intracellular species observed is indeed the complex between E,Zn-SOD1^SH^ and CCS-D2. The crosspeaks arising from SOD1 in the complex formed *in vitro* were in slow exchange with those of free SOD1 in the presence of sub-stoichiometric CCS-D2 (Supplementary Figure S2). ^15^N relaxation analysis on the complex *in vitro* resulted in a molecular reorientation correlation time (τ_m_) of 17.2 ± 1.8 ns, estimated from ^15^N R_2_/R_1_ ratios. Such value is close to that reported for the E,Zn-SOD1^SH^ homodimer (τ_m_ = 20.6 ± 0.9 ns)^13^ and is consistent with the formation of a 32 kDa heterodimer between SOD1 and CCS-D2 (the latter having essentially the same molecular weight of one SOD1 monomer).

Taken together, these data show that CCS-D2 stably interacts with WT E,Zn-SOD1^SH^ in the cytoplasm, forming a heterodimer which is preserved upon cell lysis.

### CCS-D2 interacts with and stabilizes fALS SOD1 mutants

**Figure 2:**
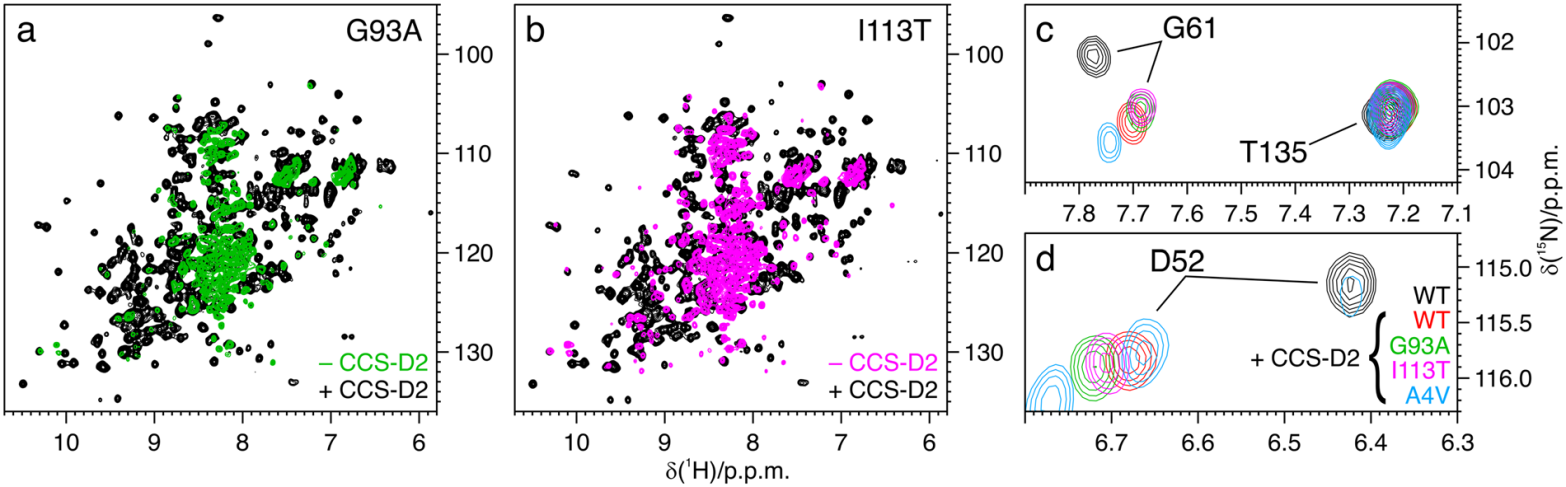
CCS-D2 stabilizes fALS SOD1 mutants and allows zinc binding. (**a**) ^1^H-^15^N NMR spectra of lysates from cells expressing [U-^15^N]-labelled G93A SOD1 alone (green) and together with [U-^15^N]-labelled CCS-D2 (black). (**b**) ^1^H-^15^N NMR spectra of lysates from cells expressing [U-^15^N]-labelled I113T SOD1 alone (magenta) and together with [U-^15^N]-labelled CCS-D2 (black). (**c**,**d**) Detailed views of characteristic SOD1 signals in the ^1^H-^15^N NMR spectra of lysates containing [U-^15^N]-labelled WT SOD1 alone (black), and WT SOD1 (red) and mutants G93A, I113T, A4V (green, magenta and azure, respectively) in the complex with CCS-D2, showing that SOD1 mutants experience chemical shift changes similar to WT SOD1 upon complex formation. See also Supplementary Figure S3.

As shown previously, some WT-L fALS SOD1 mutants do not bind zinc when overexpressed in human cells, and accumulate in the cytoplasm as unstructured species^28^. When CCS-D2 was co-expressed with G93A (100.2 ± 4.5 µM and 114.2 ± 1.7 µM, respectively) and with I113T (107.1 ± 1.6 µM and 107.4 ± 4.9 µM, respectively) SOD1 mutants, the amount of unstructured species was greatly decreased in the in-cell NMR spectra and the signals arising from the heterodimer with CCS-D2 were observed (Fig. 2a,b). Also in this case the complex was unaltered upon cell lysis, and no additional conformations of SOD1 were observed, indicating a stable interaction between mutant SOD1 and CCS-D2 (Supplementary Figure S3a,b). Chemical shift comparison with the corresponding spectra of WT SOD1, both in cells and in the lysate, indicated that the SOD1 mutants in the complex with CCS-D2 have the same conformation of WT SOD1 in the same complex, i.e. they have bound zinc (Fig. 2c,d). The only significant chemical shift differences are ascribed to the single-point mutations. Also the A4V SOD1 mutant, which is severely destabilized in the apo state, was partially stabilized by CCS-D2 (Supplementary Figure S3c). Due to the lower expression levels of A4V SOD1 (61.8 ± 2.1 µM) compared to co-expressed CCS-D2 (83.1 ± 8.5 µM) and to the other mutants, additional signals were observed in the NMR spectra of the lysate arising from an excess of free CCS-D2 homodimer (Supplementary Figure S3d).

The interaction between CCS-D2 and T54R SOD1 (80.4 ± 6.5 µM and 85.2 ± 10.0 µM, respectively) was also investigated. The T54R mutation does not cause the misfolding of SOD1, but interferes with the disulfide bond formation mediated by CCS^28^. Interestingly, T54R SOD1 also formed a stable complex with CCS-D2 (Supplementary Figure S4), indicating that the mutation did not affect the interaction with D2 (as it would be expected from the structure reported by Lamb *et al*.^18^), but likely interferes with the D3-mediated disulfide bond formation (consistent with a more recent report by Fetherolf *et al*.^24^).

Overall, these data indicate that CCS-D2 interacts with fALS SOD1 mutants in a similar fashion to the WT SOD1. CCS-D2 forms a stable heterodimer with mutant SOD1 that is structurally equivalent to the heterodimer with the zinc-bound WT SOD1. By doing so, CCS-D2 prevents the formation of the unstructured species in the cells, effectively acting as a molecular chaperone.

### CCS-D2 interacts with WT apo-SOD1^SH^*in vitro*

**Figure 3:**
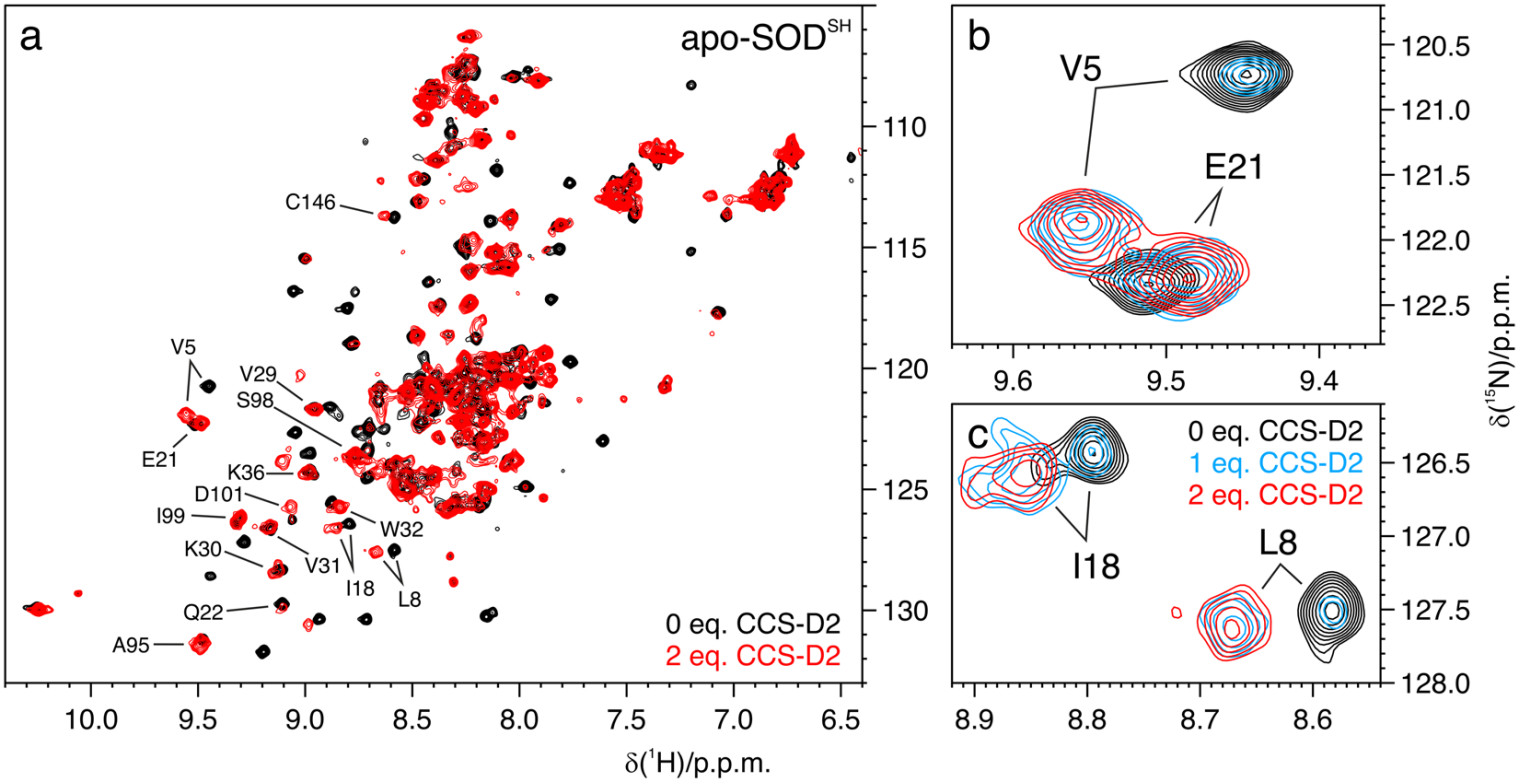
CCS-D2 interacts with apo-SOD1^SH^ *in vitro*. (**a**) *In vitro* ^1^H-^15^N NMR spectra of [U-^15^N]-labelled apo-SOD1^SH^ alone (black) and in the presence of 2 equivalents of unlabelled CCS-D2 (red). The signals used to estimate the molecular reorientation correlation time (τ_m_) of apo-SOD1^SH^ in the complex with CCS-D2 are labelled. (**b**,**c**) Detailed views of SOD1 signals (red) experiencing chemical shift changes upon titration with 1 (azure) and 2 (red) equivalents of CCS-D2, showing that apo-SOD1^SH^ free and in the complex are in the slow exchange regime.

In order to further elucidate the mechanism of action of CCS in stabilizing SOD1, we sought to determine whether CCS-D2 could also interact with the disulfide-reduced apo form of SOD1. To this aim, WT [U-^15^N]-apo-SOD1^SH^ was titrated *in vitro* with up to three equivalents of unlabelled CCS-D2 and monitored by NMR after each addition. The addition of CCS-D2 caused the disappearance of several well-dispersed NMR signals of the monomeric apo-SOD1^SH^, together with the appearance of broader peaks presumably arising from apo-SOD1^SH^ in the complex with CCS-D2 (Fig. 3a). With 1 equivalent of CCS-D2, ~20% of free apo-SOD1^SH^ was still detected; the latter disappeared only in the presence of more than 2 equivalents of CCS-D2, suggesting that its affinity for CCS-D2 is lower than that of E,Zn-SOD1^SH^ (Fig. 3b,c).

^15^N relaxation analysis performed on the final 1:3 mixture of apo-SOD1^SH^ and CCS-D2 resulted in an estimated τ_m_ of 18.6 ± 1.4 ns, obtained by analyzing a set of well-dispersed signals arising mainly from residues in the β-barrel of SOD1 (shown in Fig. 3a). Such value is comparable to that of the complex between E,Zn-SOD1^SH^ and CCS-D2, and is markedly increased from the τ_m_ reported for the monomeric apo-SOD1^SH^ (10.3 ± 0.4 ns)^13^.

In order to assess whether apo-SOD1^SH^ in the complex with CCS-D2 could bind zinc, the apo-SOD1^SH^ – CCS-D2 complex was further titrated with 1 equivalent of zinc per SOD1 monomer. The NMR spectra of the final point of the titration revealed that the E,Zn-SOD1^SH^ – CCS-D2 complex had formed (Supplementary Figure S5a), identical to that obtained by direct titration of E,Zn-SOD1^SH^ with CCS-D2 (Supplementary Figure S5b and Fig. 1c), meaning that interacting with CCS while in the apo state would eventually lead SOD1 towards the same maturation pathway observed for the zinc-bound form.

Taken together, these data are consistent with the formation of a heterodimer between apo-SOD1^SH^ and CCS-D2 with the same size as that between E,Zn-SOD1^SH^ and CCS-D2. Therefore, CCS-D2 can also bind the non-metallated form of SOD1, albeit with lower affinity, possibly contributing to the stabilization effect observed in cells, and allowing zinc binding to occur subsequently on the apo-SOD1^SH^ – CCS-D2 complex.

## Discussion

fALS-linked SOD1 mutations are known to have several detrimental effects on the folding stability and on the maturation pathway of SOD1. In particular, several wild type-like mutations have been associated to a thermodynamic destabilization of SOD1, and have been shown to decrease the conformational stability of the apo, disulfide-reduced protein^11,30^. Furthermore, some mutants have been shown to destabilize the homodimer, due to a reduced association constant for the dimer formation^31,32^. The latter effect contributes to shift the equilibrium in cell towards the monomeric state, while the former increases the rate of the formation of the unstructured species. The interaction between SOD1 and CCS – mediated by D2 – has been extensively investigated before^15–24^, and CCS is known to have a stabilizing effect on the maturation pathway of SOD1: overexpression of CCS in cell cultures and transgenic mice has previously revealed a protective function against the aggregation of mutant SOD1^33–35^. Traditionally, the ability of CCS to rescue SOD1 from unfolding and aggregation has been attributed to its metallochaperone and oxidoreductase activities (i.e. copper transfer and formation of the disulfide bond), and D2, the SOD-like domain of CCS, has been relegated to a simple recognition function, required to allow the formation of the transient SOD1-CCS complex. Here we show that CCS-D2 forms a stable complex with mutant SOD1, and by doing so it prevents the accumulation of unstructured apo-SOD1 in the cytoplasm. Our observations are consistent with the protective role of full-length CCS previously observed *in vivo* ^33–35^. Compared to SOD1, CCS is expressed at lower levels in living organisms^36^, but its functional complex with immature SOD1 is transient and leads to the formation of fully mature SOD1, allowing CCS to be recycled and interact with a new molecule of immature SOD1. Unlike full-length CCS, the complex between SOD1 and CCS-D2 obviously cannot proceed towards fully mature SOD1 and becomes the end point of the pathway. Such complex is clearly observed by in-cell NMR and corresponds to a SOD1 dimer-like heterodimer containing a SOD1 and a CCS-D2 subunit, relatively free to move in the cytosol. Notably, the SOD1 mutants in the complex with CCS-D2 have bound a zinc ion, and are structurally equivalent to WT E,Zn-SOD1^SH^ in the same complex. Therefore, the stabilizing effect of CCS-D2 on mutant SOD1 is twofold: it prevents the accumulation of unstructured apo protein and facilitates zinc binding.

**Figure 4:**
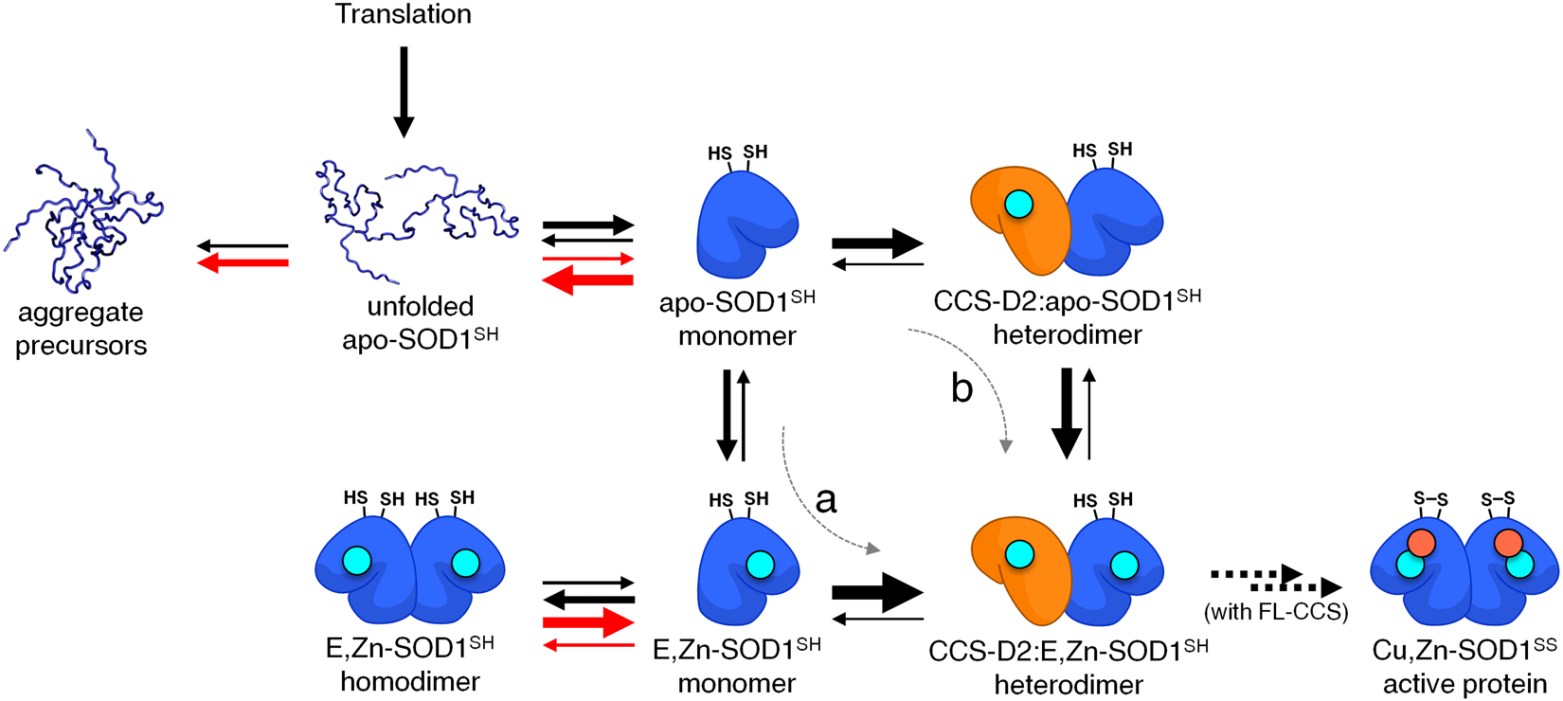
Proposed model for the molecular chaperone activity of CCS-D2. Schematic drawing of the initial maturation steps of SOD1 illustrating the role of CCS-D2 in stabilizing immature SOD1. The arrows show the preferred direction of each step. Red arrows indicate which maturation steps are negatively influenced by fALS-SOD1 mutations. Two possible mechanisms of action for CCS-D2 are shown: (**a**) SOD1 first binds zinc and then interacts with CCS-D2; (**b**) apo-SOD1 first interacts with CCS-D2 and then binds zinc. While the complex formed between SOD1 and CCS-D2 is stable and can be observed in the cells, the interaction between SOD1 and copper-bound FL-CCS (not shown) leads to the fully mature Cu,Zn-SOD1^SS^ protein.

Based on these observations and on those previously reported, we can summarize the pathway required for SOD1 maturation, and qualitatively indicate in which step CCS will positively affect the pathway (Fig. 4). We can hypothesize two possible modes of actions for the stabilizing effect of CCS-D2, which are not mutually exclusive: one is that zinc binding to SOD1 occurs first, and then CCS interacts with E,Zn-SOD1^SH^ (Fig. 4a). This pathway would be most consistent with what observed for WT SOD1, as it is known to bind zinc spontaneously in the cytoplasm. In the case of mutant SOD1 however, it would imply that the protein would bind zinc and then, in the absence of CCS, lose it.

The other mode of action is that CCS first interacts with monomeric apo-SOD1^SH^ and stabililizes it in a homodimer-like folding state, and then apo-SOD1 in the heterodimer binds zinc (Fig. 4b). In order to determine whether this mechanism occurs in the cell, we would have to separate in time the formation of the heterodimer and the zinc binding. However, this is difficult to achieve because cells have to be supplied with zinc during protein expression, due to the fact that also CCS-D2 needs zinc to fold correctly. *In vitro*, on the other hand, the two events are easily separated as zinc-containing CCS-D2 can be mixed with apo-SOD1^SH^. Our data show that CCS-D2 indeed interacts with apo-SOD1^SH^ and forms a heterodimer *in vitro*, albeit with lower affinity than with E,Zn-SOD1^SH^. The NMR signals arising from the folded part of free apo-SOD1^SH^ appeared to be in a slow-intermediate exchange regime with the corresponding signals in the complex, implying a dynamic equilibrium in the milliseconds time scale, slow enough to allow the conformational rearrangement of SOD1 as a consequence of zinc binding. While the interaction between apo-SOD1^SH^ and CCS-D2 had been reported before^22,23^, its functional role had not been fully recognized and deserves further characterization.

In this work, we showed that the SOD-like domain of CCS acts as a molecular chaperone towards immature SOD1 and helps to stabilize the folded state of fALS-linked wild type-like SOD1 mutants, favouring their correct maturation within the cell. CCS-D2-mediated stabilization has a twofold effect: it prevents the accumulation of unstructured apo species of SOD1, which are the potential precursors of pathogenic forms such as oligomers or aggregates, and allows SOD1 to bind zinc, which is a critical step of its maturation towards the enzymatically active and structurally stable protein. We showed that *in vitro* CCS-D2 can interact either with apo-SOD1^SH^ or with E,Zn-SOD1^SH^, with higher affinity for the latter, with which it produces a stable complex that is also observed in living cells. While both mechanisms would allow SOD1 to progress through the correct maturation pathway, the direct interaction between CCS and apo-SOD1^SH^ gives credit to the hypothesis that CCS stabilizes SOD1 prior to zinc binding. The molecular chaperone role of CCS described here adds further depth to our understanding of how this protein is critical for the correct maturation of SOD1, and will likely have important implications in the context of developing novel strategies for preventing the cytotoxic effects of mutant SOD1 in familial ALS.

## Methods

### Gene cloning

The cDNA encoding the SOD-like domain of human CCS (amino acids 84–234, GenBank: NP_005116.1) was amplified by PCR using the primers D2-F (5′-CCGGAATTCGCCACCATGCAGAATCTGGGGGCAGCAGTGGCC-3′) and D2-R (5′-ATAACCGCTCGAGAAGCTTTTTAAGCGGAGCGTGCAATGATGCCACAG-3′), and cloned into the pHLsec^37^ vector between EcoRI and XhoI restriction enzyme sites to generate the mammalian expression plasmid. The clone was verified by DNA sequencing. pHLsec vectors encoding WT human SOD1 (amino acids 1–154, GenBank: NP_000445.1) and the mutants A4V, T54R, G93A and I113T had been generated previously^28^.

### Human cell culture and transfection

HEK293T (ATCC CRL-3216) cells were maintained in DMEM high glucose (Life Technologies) supplemented with L-glutamine (Life Technologies, 2 mM final concentration), antibiotics (penicillin and streptomycin, Life Technologies, 100 U/ml final concentration) and 10% FBS (Gibco) in uncoated 75 cm^2^ plastic flasks and incubated at 37 °C, 5% CO_2_ in a humidified atmosphere. Cells were transiently transfected with the pHLsec plasmid containing the gene of interest using polyethylenimine (PEI, branched, average M_w_ 25 kDa, Sigma-Aldrich), as previously described^38^. For co-expression of SOD1 (both WT and mutants) and CCS-D2, cells were transfected with plasmids containing the constructs in different amounts and ratios. The final ratio was chosen in order to obtain similar expression levels of the two proteins; specifically a weight ratio of 1:1:2 (25 µg SOD1 WT or mutant DNA; 25 µg CCS-D2 DNA; 50 µg PEI per 75 cm^2^ flask) was used. [U-^15^N]-BioExpress6000 medium (Cambridge Isotope Laboratories) was used for in-cell NMR samples, supplemented with 2% FBS, antibiotics and 10 µM of ZnSO_4_.

### In-cell NMR sample preparation

Samples for in-cell NMR were prepared following a reported protocol^2^. Briefly, transfected cells were detached with trypsin, suspended in DMEM + 10% FBS, washed once with PBS and re-suspended in one pellet volume of DMEM supplemented with 90 mM glucose, 70 mM HEPES and 20% D_2_O. The cell suspension was transferred in a 3 mm Shigemi NMR tube, which was gently spun to sediment the cells at the bottom. Cell viability before and after NMR experiments was assessed by trypan blue staining. After the NMR experiments, the cells were collected and the supernatant was checked for protein leakage by NMR. The cell lysates were prepared by freeze-thaw cycles in PBS buffer followed by centrifugation to remove the insoluble fraction. The supernatant was collected for NMR analysis.

### Expression and purification of SOD1

WT SOD1 protein was prepared following an existing protocol^39^. Briefly, a cell culture of *E*. *Coli* BL21(DE3) Gold (Stratagene), transformed with a pET28a plasmid containing the WT SOD1 gene was grown overnight at 37 °C in LB, harvested and re-suspended in ^15^N-labelled M9 medium. ZnSO_4_ was added in the culture to a final concentration of 100 µM. After 4 h from induction with 0.5 mM IPTG at 30 °C the cells were harvested and re-suspended in 20 mM Tris, pH 8 buffer for lysis. The cleared lysate was loaded on an anion exchange column (HiPrep Q FF 16/10, GE Healthcare) for a first purification by elution with NaCl gradient. The fractions containing SOD1 (checked by SDS-PAGE) were collected and further purified by gel filtration (Superdex 75 26/60 column, GE Healthcare) in 20 mM Tris, 100 mM NaCl, pH 8 buffer. Fractions containing pure SOD1 were collected. 1 mM DTT was added in all buffers to prevent protein aggregation mediated by disulfide bonds.

Apo-SOD1^SS^ was produced by repeated dialysis against 10 mM EDTA in 50 mM acetic acid at pH 3.5. Then, the buffer was exchanged in phosphate-buffered saline (PBS) pH 7.4. To obtain apo-SOD1^SH^, apo-SOD1^SS^ was incubated 40 min at 37 °C with 50 mM of DTT. Following disulfide bond reduction, the buffer was exchanged to remove DTT and dissolved oxygen. E,Zn-SOD1^SH^ was then obtained by adding one equivalent per monomer of ZnSO_4_ in anaerobic conditions. Correct disulfide reduction and metallation were checked by ^1^H-^15^N NMR.

### Expression and purification of CCS-D2

The synthetic gene encoding CCS-D2 cloned in the pTH34 plasmid with an N-terminal histidine tag was obtained previously^22^. The protein was expressed in *E*. *coli* BL21(DE3) C41 cells (Stratagene). Protein expression was induced with 0.7 mM IPTG for 16 h at 30 °C. ZnSO_4_ was added in the culture to a final concentration of 100 µM. Purification was performed using a nickel-chelating HiTrap (GE Healthcare) column. After digestion with AcTEV protease (Invitrogen) O/N at 25 °C the protein was separated from the affinity tag in a HiTrap column. To further separate CCS-D2 from the residual affinity tag, the digested protein was loaded on a Superdex 75 26/60 (GE Healthcare) chromatographic column and the fraction containing pure CCS-D2 were collected. The buffer was then exchanged in anaerobic conditions to remove dissolved oxygen.

### NMR experiments

In-cell NMR spectra were collected at 308 K at a 950 MHz Bruker Avance III spectrometer equipped with a TCI CryoProbe. For each cell sample, a 2D ^1^H-^15^N SOFAST-HMQC^40^ spectrum was recorded with 64 scans, 2048 points, 128 increments and a 0.3 s recycle delay (duration ~1 h). NMR spectra of the corresponding cell lysates were collected at 950 MHz with the same experimental parameters.

*In vitro* titration of WT [U-^15^N]-E,Zn-SOD1^SH^ with CCS-D2 was performed at 950 MHz on a sample of 140 µM E,Zn-SOD1^SH^ in PBS buffer (pH 7.4). Increasing amounts of CCS-D2 in the same buffer were added in anaerobic conditions up to 1 equivalent, and 2D ^1^H-^15^N SOFAST-HMQC spectra were acquired at 308 K after each addition. *In vitro* titration of WT [U-^15^N]-apo-SOD1^SH^ with CCS-D2 was performed at a 900 MHz Bruker Avance III spectrometer equipped with a TCI CryoProbe. Increasing amounts of CCS-D2 were added to a sample of 100 µM apo-SOD1^SH^ in PBS buffer (pH 7.4) under anaerobic conditions, up to three equivalents. 2D ^1^H-^15^N SOFAST-HMQC spectra were acquired after each addition at 298 K.

*In vitro* ^15^N-R_1_, R_2_ relaxation experiments were collected on both E,Zn-SOD1^SH^ and apo-SOD1^SH^ – CCS-D2 complexes at 298 K at a 500 MHz Bruker spectrometer equipped with a TCI CryoProbe. The ^15^N-R_1_, R_2_ values for each crosspeak were obtained by fitting the signal intensity at increasing evolution times with mono-exponential decays in OriginPro. The molecular reorientation correlation times (τ_m_) for both complexes was estimated from the averaged R_2_/R_1_ ratio of a subset of well-dispersed crosspeaks that were known to arise from the folded part of the protein and exhibited homogeneous relaxation properties.

All the NMR spectra collected were processed with Bruker Topspin NMR data-processing software. The in-cell NMR spectra were further processed by subtracting a spectrum of cells transfected with empty vector, acquired in the same experimental conditions, to eliminate the signals arising from partial ^15^N labelling of other cellular components.

### Protein quantification

The expression levels of WT/mutant SOD1 and CCS-D2 were determined by Coomassie-stained SDS-PAGE (Supplementary Figure S6). Lysates from cell samples co-expressing SOD1 and CCS-D2 were run at increasing dilutions together with purified WT SOD1 and CCS-D2 as references. Densitometry analysis was performed with ImageJ. The values reported in the main text reflect the protein concentrations calculated in the cell lysates, which correspond to the effective concentrations in the in-cell NMR samples (mean value ± S.E.M.).

### Data Availability

All the data generated and analysed during the current study are available from the corresponding author on reasonable request.

## Electronic supplementary material

Supplementary Information

## References

[1] Rosen DR (1993). Mutations in Cu/Zn superoxide dismutase gene are associated with familial amyotrophic lateral sclerosis. Nature.

[2] Kaur SJ, McKeown SR, Rashid S (2016). Mutant SOD1 mediated pathogenesis of Amyotrophic Lateral Sclerosis. Gene.

[3] Shibata N (1996). Intense superoxide dismutase-1 immunoreactivity in intracytoplasmic hyaline inclusions of familial amyotrophic lateral sclerosis with posterior column involvement. J. Neuropathol. Exp. Neurol..

[4] Watanabe M (2001). Histological evidence of protein aggregation in mutant SOD1 transgenic mice and in amyotrophic lateral sclerosis neural tissues. Neurobiol. Dis..

[5] Seetharaman SV (2009). Immature Copper-Zinc Superoxide Dismutase and FamilialAmyotrophic Lateral Sclerosis. Exp. Biol. Med. Maywood NJ.

[6] Lelie HL (2011). Copper and zinc metallation status of copper-zinc superoxide dismutase from amyotrophic lateral sclerosis transgenic mice. J. Biol. Chem..

[7] Banci L (2007). Metal-free superoxide dismutase forms soluble oligomers under physiological conditions: a possible general mechanism for familial ALS. Proc. Natl. Acad. Sci. USA.

[8] Furukawa Y, Kaneko K, Yamanaka K, O’Halloran TV, Nukina N (2008). Complete loss of post-translational modifications triggers fibrillar aggregation of SOD1 in the familial form of amyotrophic lateral sclerosis. J. Biol. Chem..

[9] Hayward LJ (2002). Decreased metallation and activity in subsets of mutant superoxide dismutases associated with familial amyotrophic lateral sclerosis. J. Biol. Chem..

[10] Banci L (2007). Metalation of the amyotrophic lateral sclerosis mutant glycine 37 to arginine superoxide dismutase (SOD1) apoprotein restores its structural and dynamical properties in solution to those of metalated wild-type SOD1. Biochemistry.

[11] Lindberg MJ, Tibell L, Oliveberg M (2002). Common denominator of Cu/Zn superoxide dismutase mutants associated with amyotrophic lateral sclerosis: decreased stability of the apo state. Proc. Natl. Acad. Sci. USA.

[12] Oztug Durer ZA (2009). Loss of metal ions, disulfide reduction and mutations related to familial ALS promote formation of amyloid-like aggregates from superoxide dismutase. PloS One.

[13] Arnesano F (2004). The unusually stable quaternary structure of human Cu,Zn-superoxide dismutase 1 is controlled by both metal occupancy and disulfide status. J. Biol. Chem..

[14] Banci L, Bertini I, Cantini F, D’Amelio N, Gaggelli E (2006). Human SOD1 before harboring the catalytic metal: solution structure of copper-depleted, disulfide-reduced form. J. Biol. Chem..

[15] Casareno RL, Waggoner D, Gitlin JD (1998). The copper chaperone CCS directly interacts with copper/zinc superoxide dismutase. J. Biol. Chem..

[16] Schmidt PJ (1999). Multiple protein domains contribute to the action of the copper chaperone for superoxide dismutase. J. Biol. Chem..

[17] Lamb AL, Torres AS, O’Halloran TV, Rosenzweig AC (2000). Heterodimer formation between superoxide dismutase and its copper chaperone. Biochemistry.

[18] Lamb AL, Torres AS, O’Halloran TV, Rosenzweig AC (2001). Heterodimeric structure of superoxide dismutase in complex with its metallochaperone. Nat. Struct. Biol..

[19] Furukawa Y, Torres AS, O’Halloran TV (2004). Oxygen-induced maturation of SOD1: a key role for disulfide formation by the copper chaperone CCS. EMBO J..

[20] Culotta VC, Yang M, O’Halloran TV (1763). Activation of superoxide dismutases: putting the metal to the pedal. Biochim. Biophys. Acta.

[21] Leitch JM, Yick PJ, Culotta VC (2009). The right to choose: multiple pathways for activating copper,zinc superoxide dismutase. J. Biol. Chem..

[22] Banci L (2012). Human superoxide dismutase 1 (hSOD1) maturation through interaction with human copper chaperone for SOD1 (hCCS). Proc. Natl. Acad. Sci. USA.

[23] Wright GSA, Antonyuk SV, Hasnain SS (2016). A faulty interaction between SOD1 and hCCS in neurodegenerative disease. Sci. Rep..

[24] Fetherolf MM (2017). Copper-zinc superoxide dismutase is activated through a sulfenic acid intermediate at a copper ion entry site. J. Biol. Chem..

[25] Banci L (2013). Atomic-resolution monitoring of protein maturation in live human cells by NMR. Nat. Chem. Biol..

[26] Luchinat E, Banci L (2017). In-cell NMR: a topical review. IUCrJ.

[27] Plitzko JM, Schuler B, Selenko P (2017). Structural Biology outside the box-inside the cell. Curr. Opin. Struct. Biol..

[28] Luchinat E (2014). In-cell NMR reveals potential precursor of toxic species from SOD1 fALS mutants. Nat. Commun..

[29] Pope CR, De Feo CJ, Unger VM (2013). Cellular distribution of copper to superoxide dismutase involves scaffolding by membranes. Proc. Natl. Acad. Sci. USA.

[30] Lindberg MJ, Byström R, Boknäs N, Andersen PM, Oliveberg M (2005). Systematically perturbed folding patterns of amyotrophic lateral sclerosis (ALS)-associated SOD1 mutants. Proc. Natl. Acad. Sci. USA.

[31] Hough MA (2004). Dimer destabilization in superoxide dismutase may result in disease-causing properties: structures of motor neuron disease mutants. Proc. Natl. Acad. Sci. USA.

[32] Vassall KA, Stathopulos PB, Rumfeldt JAO, Lepock JR, Meiering EM (2006). Equilibrium thermodynamic analysis of amyotrophic lateral sclerosis-associated mutant apo Cu,Zn superoxide dismutases. Biochemistry.

[33] Proescher JB, Son M, Elliott JL, Culotta VC (2008). Biological effects of CCS in the absence of SOD1 enzyme activation: implications for disease in a mouse model for ALS. Hum. Mol. Genet..

[34] Cozzolino M (2009). Oligomerization of mutant SOD1 in mitochondria of motoneuronal cells drives mitochondrial damage and cell toxicity. Antioxid. Redox Signal..

[35] Winkler DD (2009). Structural and biophysical properties of the pathogenic SOD1 variant H46R/H48Q. Biochemistry.

[36] Rothstein JD (1999). The copper chaperone CCS is abundant in neurons and astrocytes in human and rodent brain. J. Neurochem..

[37] Aricescu AR, Lu W, Jones EY (2006). A time- and cost-efficient system for high-level protein production in mammalian cells. Acta Crystallogr. D Biol. Crystallogr..

[38] Seiradake E, Zhao Y, Lu W, Aricescu AR, Jones EY (2015). Production of cell surface and secreted glycoproteins in mammalian cells. *Methods Mol*. Biol. Clifton NJ.

[39] Banci L (1998). Solution structure of reduced monomeric Q133M2 copper, zinc superoxide dismutase (SOD). Why is SOD a dimeric enzyme?. Biochemistry.

[40] Schanda P, Brutscher B (2005). Very fast two-dimensional NMR spectroscopy for real-time investigation of dynamic events in proteins on the time scale of seconds. J. Am. Chem. Soc..

