# Supplementary material for: A molecular chaperone activity of CCS restores the maturation of SOD1 fALS mutants
Source: Sci Rep. 2017 Dec 12;7:17433. doi: 10.1038/s41598-017-17815-y (PMC5727297; doi:10.1038/s41598-017-17815-y)
Supplement: Supplementary file 1 — Supplementary Information [file 41598_2017_17815_MOESM1_ESM.pdf]

Supplementary Information

A molecular chaperone activity of CCS restores the maturation of SOD1 fALS mutants

Enrico Luchinat[1,2], Letizia Barbieri[1,3], Lucia Banci[1,4]*

1. Magnetic Resonance Centre (CERM), University of Florence, 50019 Sesto Fiorentino, Italy.
2. Department of Biomedical, Clinical and Experimental Sciences, University of Florence, 50134 Florence, Italy.
3. Interuniversity Consortium for Magnetic Resonance of Metallo Proteins (CIRMMP), 50019 Sesto Fiorentino, Italy.
4. Department of Chemistry, University of Florence, 50019 Sesto Fiorentino, Florence, Italy.

Supplementary Figures

Supplementary Figure S1

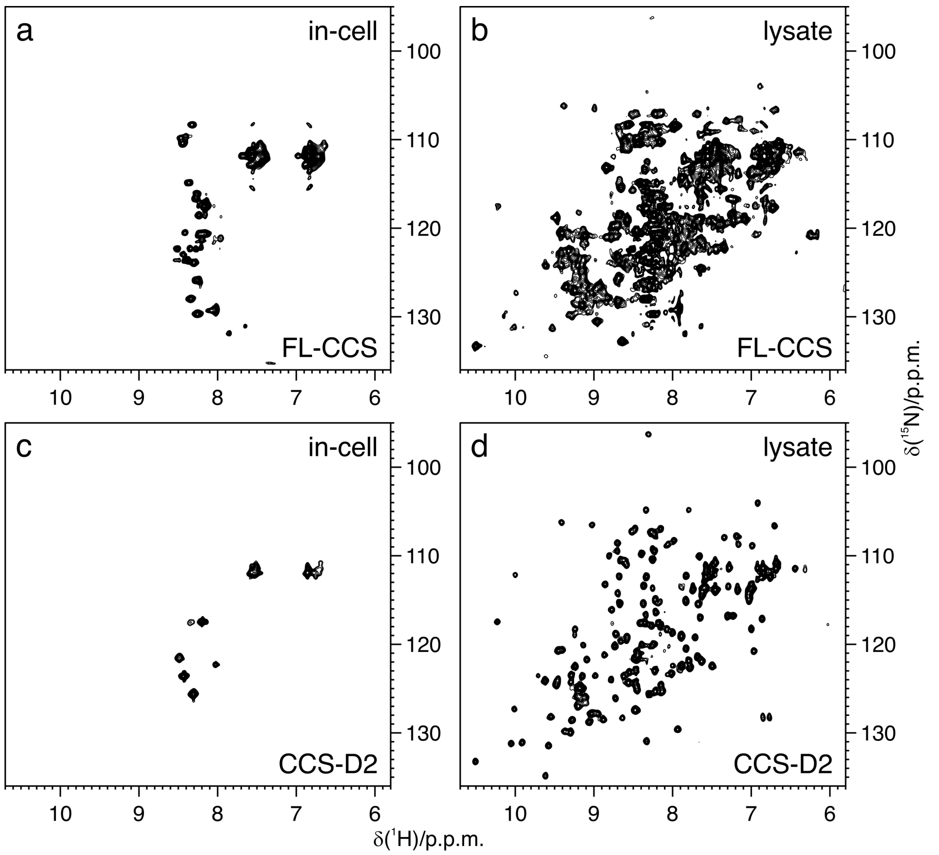

Supplementary Figure S1. Full-length CCS and CCS-D2 interact with other cellular components. (a) ^{1}H-^{15}N NMR spectrum of human cells expressing [U-^{15}N]-labelled FL-CCS; (b) ^{1}H-^{15}N NMR spectrum of the corresponding cell lysate. (c) ^{1}H-^{15}N NMR spectrum of human cells expressing [U-^{15}N]-labelled CCS-D; (d) ^{1}H-^{15}N NMR spectrum of the corresponding cell lysate.

Supplementary Figure S2

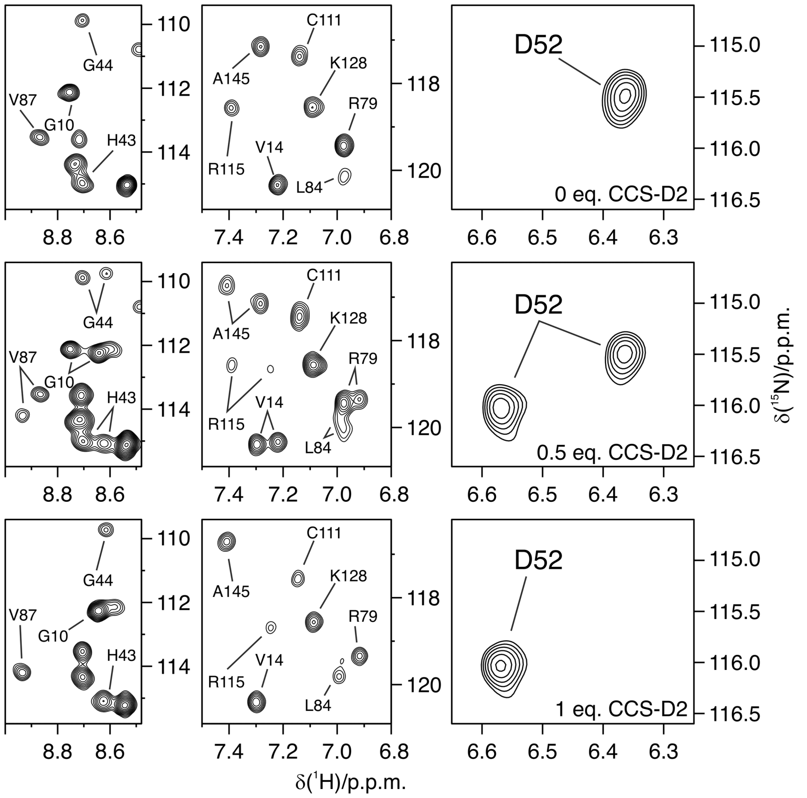

Supplementary Figure S2. Free SOD1 is in slow exchange with the complex with CCS-D2. Detailed views of ^{1}H-^{15}N *in vitro* NMR spectra of [U-^{15}N]-labelled E,Zn-SOD1^{SH} in the presence of 0 (upper row), 0.5 (middle row) and 1 (lower row) equivalents of unlabelled CCS-D2. The signal intensities in the middle row are doubled for clarity. SOD1 signals experiencing chemical shift changes upon complex formation are labelled.

Supplementary Figure S3

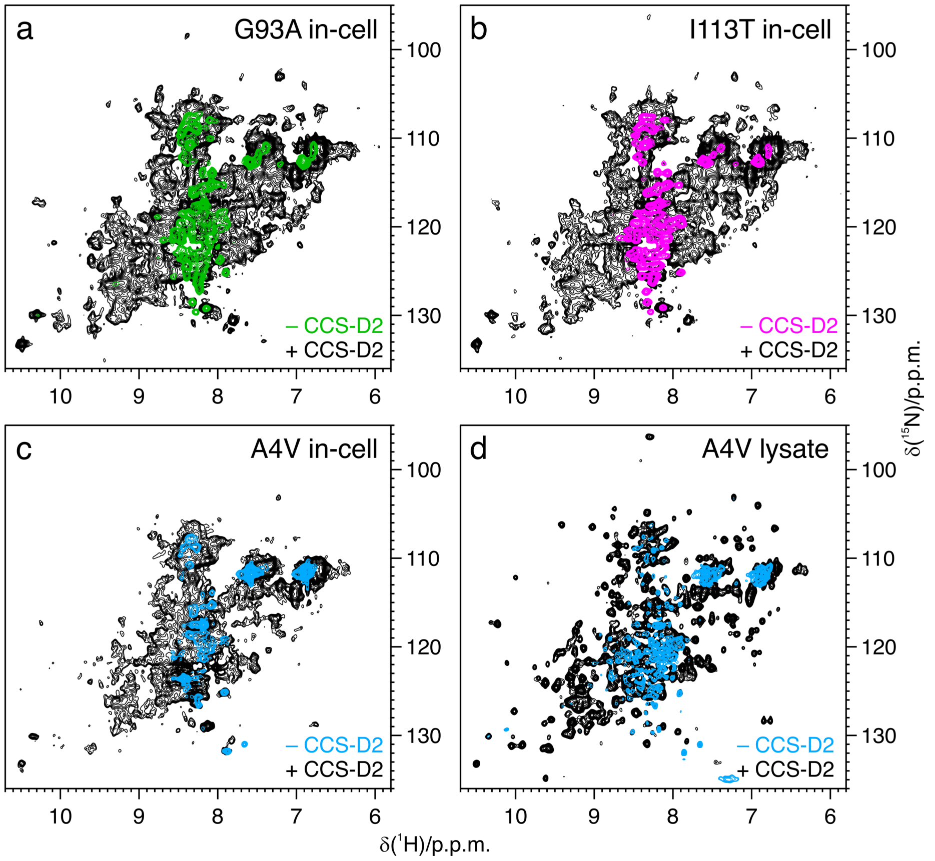

Supplementary Figure S3. ^{1}H-^{15}N NMR spectra of cells expressing [U-^{15}N]-labelled mutant SOD1 either alone or in the presence of [U-^{15}N]-labelled CCS-D2. Spectra of cells expressing (a) G93A SOD1 alone (green) and together with CCS-D2 (black); (b) I113T SOD1 alone (magenta) and together with CCS-D2 (black); (c) A4V SOD1 alone (light blue) and together with CCS-D2 (black). (d) Spectra of the lysates corresponding to the cells in (c).

Supplementary Figure S4

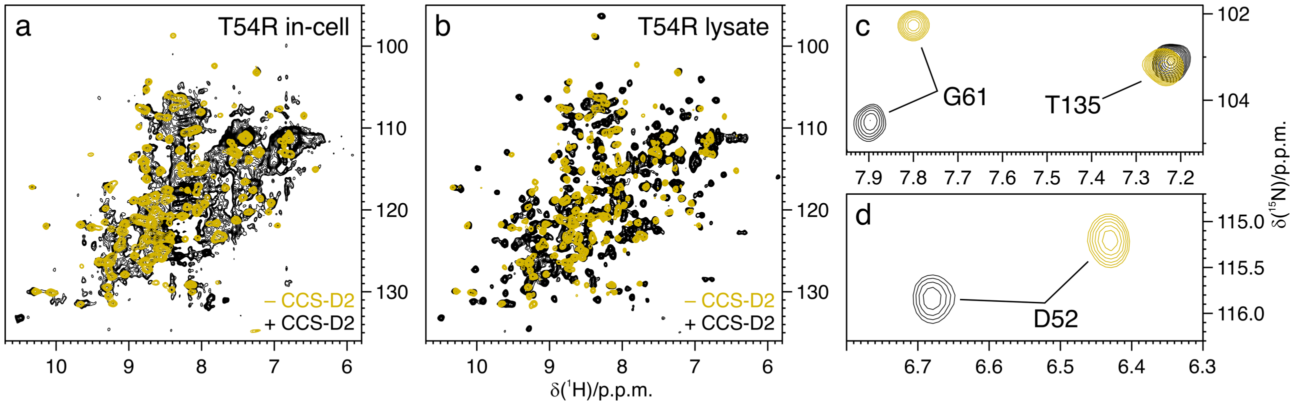

Supplementary Figure S4. CCS-D2 forms a stable complex with the SOD1 interface mutant T54R. (a) ^{1}H-^{15}N NMR spectra of cells expressing [U-^{15}N]-labelled T54R SOD1 alone (yellow) or together with [U-^{15}N]-labelled CCS-D2 (black); (b) spectra of the corresponding cell lysates; (c,d) detailed views of SOD1 signals in the ^{1}H-^{15}N NMR spectra of lysates containing T54R SOD1 alone (yellow), and in the complex with CCS-D2 (black), showing characteristic chemical shift changes upon complex formation.

Supplementary Figure S5

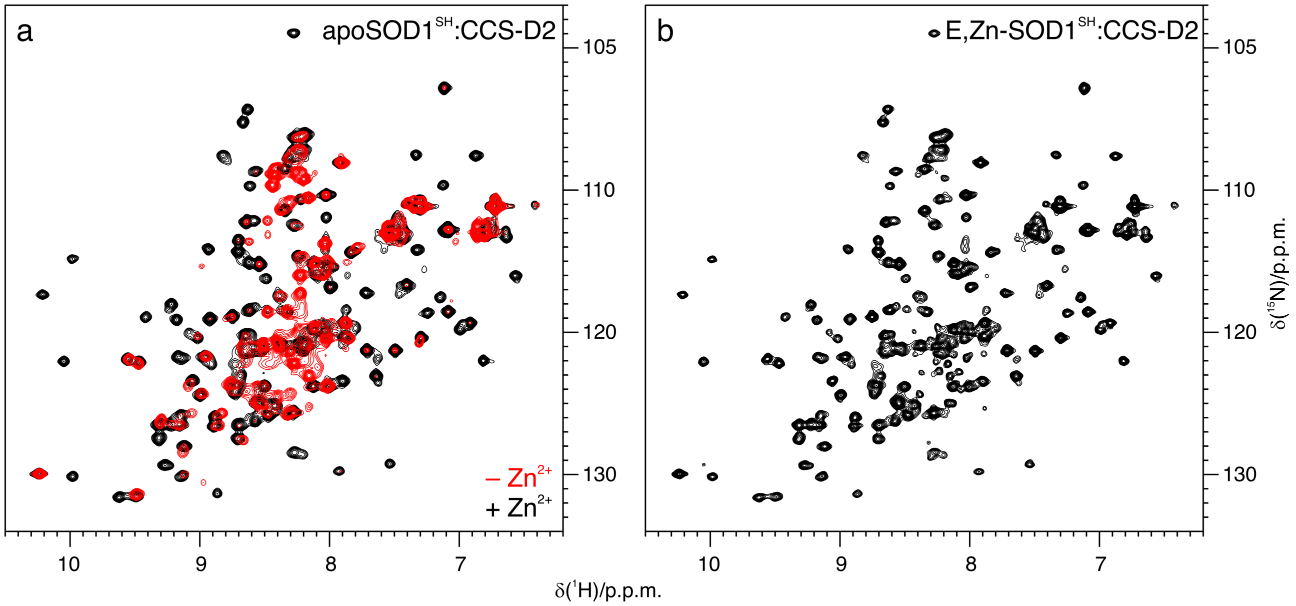

Supplementary Figure S5. apo-SOD1^{SH} in the complex with CCS-D2 binds zinc *in vitro*. *In vitro* ^{1}H-^{15}N NMR spectra of (a) [U-^{15}N]-labelled apo-SOD1^{SH} in the complex with unlabelled CCS-D2 before (red) and after titration with 1 equivalent of zinc per SOD1 monomer (black); (b) [U-^{15}N]-labelled E,Zn-SOD1^{SH} in the complex with unlabelled CCS-D2 (sample identically prepared to that shown in Figure 1c, blue), showing identical chemical shifts to those of panel (a), black.

Supplementary Figure S6

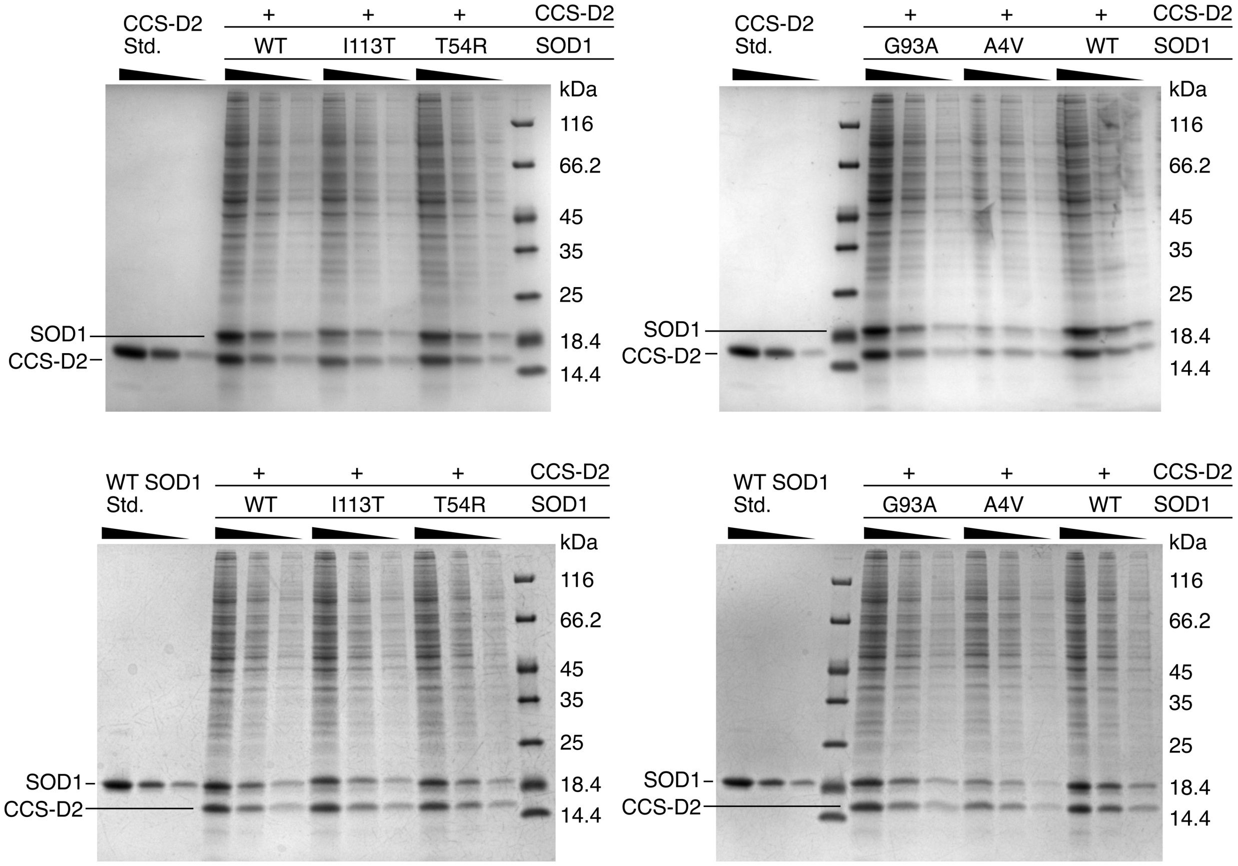

Supplementary Figure S6. Expression levels of SOD1 WT and mutants co-expressed with CCS-D2. Coomassie-stained SDS-PAGE of cell lysates co-expressing SOD1 and CCS-D2 run at increasing dilutions (from left to right, 1/15, 1/30, 1/75). Protein bands and reference molecular weights are indicated. Protein concentrations were estimated by comparing band intensities with dilutions of pure CCS-D2 (from left to right, 15, 7.5, 3 μM) and WT SOD1 (from left to right, 10, 5, 2 μM).
